# Clinical Characteristics of Children With Precocious Puberty at Al Ahsa: A Retrospective Study

**DOI:** 10.7759/cureus.117622

**Published:** 2026-10-08

**Authors:** Zainab Y AlDaif, Ahmed M Alhamrani, Yassin M Alsaleh, Zainab H Alzein, Rahmah H Alabdallah, Zainab H Buzaid, Jenan A AlGhafli, Mohammed F Al Hani, Salsabeel M AlSultan, Jumanah M Almajed, Khawla A Al Hassan

**Affiliations:** 1 Paediatrics, Maternity and Children's Hospital, Al Ahsa, SAU; 2 Paediatrics, Royal Commission Hospital, Jubail, SAU; 3 Paediatrics, King Faisal University, Al Ahsa, SAU

**Keywords:** bone age, brain mri, central precocious puberty, precocious puberty, saudi arabia

## Abstract

Background: Precocious puberty is a pediatric endocrine disorder characterized by the premature onset and progression of pubertal development. It may lead to accelerated growth, advanced bone age, early epiphyseal fusion, psychosocial concerns, and reduced adult height potential. Local data from Saudi Arabia remain limited.

Objective: This study aimed to describe the recorded clinical characteristics, diagnostic evaluation, and management patterns of children with precocious puberty evaluated at a pediatric endocrinology clinic in Al Ahsa, Saudi Arabia.

Methods: This retrospective observational study was conducted at Maternity and Children Hospital, Al Ahsa, Saudi Arabia, between 2022 and 2025. Children referred to or followed up for suspected or confirmed precocious puberty were included. Data collected included age at onset, age at diagnosis, sex, BMI category, family history, parental consanguinity, type of precocious puberty, clinical features, bone age status, brain MRI findings, and management approach. Data were summarized using descriptive statistics.

Results: A total of 87 children were included. The mean age at diagnosis was 7.4 ± 2.1 years, and the mean age at onset was 6.2 ± 1.9 years. Females accounted for 74 patients (85.1%). Central precocious puberty was the predominant type, identified in 82 patients (94.3%), while peripheral precocious puberty was documented in five patients (5.7%). Overall, 49 patients (56.3%) were overweight or obese. The most common clinical findings were breast development in 74 patients (85.1%), pubic hair development in 61 patients (70.1%), and accelerated growth velocity in 56 patients (64.4%). Advanced bone age was documented in 36 patients (41.4%). Brain MRI was abnormal in seven patients (8.0%), with no hypothalamic-pituitary or suprasellar mass lesion identified. GnRH analogue therapy was prescribed in 83 patients (95.4%).

Conclusion: Precocious puberty in this cohort was predominantly central and more common among girls. Overweight and obesity were frequent, and most imaged patients had normal brain MRI findings. These findings provide local data and support the need for standardized evaluation and multicenter studies in Saudi Arabia.

## Introduction

Precocious puberty is defined as the development of secondary sexual characteristics before eight years of age in girls and nine years of age in boys [1,2]. It is one of the most common disorders of pubertal timing encountered in pediatric endocrinology and should be differentiated from benign pubertal variants such as premature thelarche, premature adrenarche, and nonprogressive early pubertal signs [2]. Clinically, true precocious puberty is suggested by progressive pubertal development, accelerated growth velocity, advanced bone age, and biochemical evidence of pubertal activation [1,3]. Precocious puberty is broadly classified into central precocious puberty (CPP), which is gonadotropin-dependent and results from premature activation of the hypothalamic-pituitary-gonadal axis, and peripheral precocious puberty, which is gonadotropin-independent and results from excess sex steroid production from gonadal, adrenal, exogenous, or tumor-related sources [1,3]. The causes of precocious puberty vary according to sex, age, pubertal pattern, and population characteristics. Idiopathic CPP is the most common cause in girls, whereas boys and very young girls are more likely to have an underlying organic pathology such as central nervous system (CNS) lesions, congenital malformations, prior cranial irradiation, or hypothalamic hamartoma [1,3]. Peripheral causes include congenital adrenal hyperplasia, McCune-Albright syndrome, ovarian cysts or tumors, testicular tumors, adrenal tumors, severe primary hypothyroidism, familial male-limited precocious puberty, and exposure to exogenous sex steroids [1,3]. The diagnostic approach depends on clinical progression, auxological assessment, Tanner staging, bone age, basal or stimulated gonadotropins, sex steroid levels, thyroid function, adrenal androgen profile when indicated, pelvic ultrasonography in girls, and brain magnetic resonance imaging in selected patients [1,3,4]. Management depends on the underlying mechanism. Gonadotropin-releasing hormone analogues are the standard therapy for progressive CPP, aiming to slow pubertal progression, preserve adult height potential, delay early menarche when appropriate, and reduce psychosocial burden [4-6]. In peripheral precocious puberty, treatment should target the underlying source of sex steroid excess [1,3].

The consequences of untreated progressive precocious puberty include rapid skeletal maturation, early epiphyseal fusion, compromised final adult height, early menarche, and psychosocial difficulties related to body image, emotional distress, and mismatch with peers [1,5,6]. Although the psychosocial impact varies between studies, recent data from the Arab region showed lower health-related quality-of-life scores among children with CPP compared with controls, with improvement after one year of gonadotropin-releasing hormone analogue therapy [7]. These findings emphasize the importance of early recognition, appropriate evaluation, and individualized treatment. Globally, reported estimates of precocious puberty vary widely because studies differ in case definitions, age cutoffs, ascertainment methods, and whether they include CPP only or all forms of precocious puberty [8]. A recent systematic review and meta-analysis reported pooled survey-based prevalence estimates of 7.87% among girls and 3.98% among boys, whereas registry-based studies reported lower but highly variable prevalence rates ranging from 37 to 935.1 per 100,000 girls and 0.46 to 37.4 per 100,000 boys. Incidence estimates also varied substantially, ranging from 1.123 to 489.3 per 100,000 girls and 0.096 to 22.4 per 100,000 boys [8]. This heterogeneity may reflect differences in methodology, ethnicity, nutrition, obesity, socioeconomic factors, environmental exposures, and access to healthcare [8,9]. Recent evidence has also associated precocious puberty with higher body mass index (BMI), maternal age at menarche, breastfeeding duration, and hormonal markers such as luteinizing hormone, follicle-stimulating hormone, and estradiol [9].

In Arab and Gulf countries, population-based prevalence data on precocious puberty remain limited. Most available studies are hospital-based or focused on selected cohorts, quality of life, awareness, or normal pubertal timing rather than true population prevalence [7,10-14]. A Sudanese pediatric endocrinology center experience described CPP over 10 years [10], while an Egyptian study reported impaired quality of life among children with CPP [7]. In the Gulf region, published data remain sparse, and available literature more commonly addresses pubertal timing, community awareness, or related endocrine outcomes rather than the full epidemiology of precocious puberty [11-14]. This gap is relevant because regional differences in genetic background, obesity burden, environmental exposures, referral patterns, and healthcare access may influence the clinical spectrum of precocious puberty [8,9]. In Saudi Arabia, data on precocious puberty remain insufficient. A retrospective experience from a major teaching hospital in central Saudi Arabia reported 62 patients with precocious puberty over more than two decades; 43 had CPP and 19 had peripheral precocious puberty, with idiopathic CPP being the most common pattern among girls [11]. However, this was a hospital-based study and cannot be interpreted as a national prevalence estimate [11]. Saudi studies on normal pubertal timing have provided useful local reference data [12,13], and a recent Cureus study from Riyadh reported limited community awareness regarding precocious puberty and its complications [14]. These findings highlight the need for additional local studies describing the clinical presentation, etiological profile, diagnostic findings, and management patterns of precocious puberty among Saudi children.

Therefore, the present study was conducted to describe the recorded clinical characteristics of children with precocious puberty evaluated at our center. The study also aimed to characterize their demographic profile, pubertal presentation, documented diagnostic evaluation, etiological classification, and management patterns.

## Materials and methods

This retrospective observational study was conducted at Maternity and Children Hospital, Al Ahsa, Saudi Arabia, between 2022 and 2025. The study aimed to describe the clinical characteristics of precocious puberty among children referred to or followed up in the pediatric endocrinology clinic. The primary objective was to describe the demographic and clinical characteristics of children with precocious puberty evaluated in the clinic during the study period. The study population included pediatric patients referred to or followed up in the pediatric endocrinology clinic for suspected or confirmed precocious puberty. Precocious puberty was defined as the development of secondary sexual characteristics before eight years of age in girls and before nine years of age in boys [1,2]. Patients were classified according to documented clinical, biochemical, and radiological assessments as CPP, peripheral precocious puberty, or other diagnostic categories when applicable. CPP was considered when there was evidence of hypothalamic-pituitary-gonadal axis activation, whereas peripheral precocious puberty was considered when pubertal development was independent of central gonadotropin activation.

The classification of central or peripheral precocious puberty was based on the final diagnosis documented by the treating pediatric endocrinologist after integration of the clinical presentation, pubertal progression, growth pattern, available biochemical evaluation, bone age assessment, and imaging findings when indicated. CPP was considered when the clinical record documented activation of the hypothalamic-pituitary-gonadal axis, whereas peripheral precocious puberty was considered when pubertal development was documented to occur independently of central gonadotropin activation [1,3]. Because this was a retrospective chart review, hormonal testing was not prospectively standardized for research purposes, and individual basal or stimulated LH, FSH, estradiol, and testosterone values were not consistently available in an extractable format across the entire cohort. Consequently, the investigators did not retrospectively reclassify patients using a single biochemical cutoff; rather, the documented pediatric endocrinology diagnosis was used for study classification. This limitation was considered when interpreting the diagnostic and treatment findings.

All eligible patients identified during the predefined study period were included using a total enumeration approach. Therefore, the sample size was determined by the number of eligible medical records available during the study period rather than by an a priori power calculation or margin-of-error estimate. This approach was chosen because the study was descriptive and intended to characterize all available eligible cases at the study center rather than estimate population prevalence or test a prespecified hypothesis. A total of 87 patients fulfilled the eligibility criteria and were included. The relatively small cohort, particularly the small numbers of male patients and peripheral precocious puberty cases, limits the ability to characterize uncommon manifestations and was considered a study limitation.

Eligibility was determined by age at pubertal onset rather than age at eventual diagnosis. Girls were eligible when pubertal development began before eight years of age and boys when it began before nine years of age. Accordingly, patients remained eligible if the medical record documented qualifying early pubertal onset despite delayed referral or delayed formal diagnosis. This explains the maximum recorded age at diagnosis of 16 years in the cohort. Patients were excluded if their records were incomplete or unavailable, if the documented presentation represented normal pubertal timing or a benign nonprogressive pubertal variant without precocious puberty, if the age at pubertal onset did not meet the eligibility definition, or if duplicate records were identified.

Data collection

Data were retrospectively abstracted from the available medical records using a structured data collection sheet. Source records included pediatric endocrinology clinic documentation, anthropometric and growth records, available laboratory records, radiology reports, and medication or treatment documentation. The collected variables included age at diagnosis, age at pubertal onset, age group at diagnosis, sex, BMI, BMI category, family history of precocious puberty, parental consanguinity, documented type of precocious puberty, recorded clinical pubertal features at presentation, bone age status, brain MRI status, and management approach. Data completeness was assessed for each variable, and unavailable information was retained as not documented rather than imputed.

BMI was calculated as weight in kilograms divided by height in meters squared and categorized using age- and sex-specific BMI percentiles. According to the Centers for Disease Control and Prevention, BMI was categorized as underweight below the 5th percentile, healthy weight from the 5th to less than the 85th percentile, overweight from the 85th to less than the 95th percentile, and obesity at or above the 95th percentile [15]. Bone age status was extracted from the radiology report or pediatric endocrinology documentation as advanced, normal/appropriate for chronological age, or not documented. Bone age images were not independently re-read for this study, and the specific atlas or numerical threshold used to define advancement was not consistently documented in the retrospective records; therefore, no additional study-specific cutoff was retrospectively imposed. Brain MRI was similarly performed as part of routine clinical care rather than according to a study-specific imaging protocol. MRI status and findings were extracted from the clinical and radiology records, and decisions to perform imaging had been made by the treating clinical team according to the individual clinical context. The management variable was restricted to the categories actually observed in the study dataset: GnRH analogue therapy and observation/no intervention. Height-for-age z-scores, predicted adult height, and final adult height were not predefined variables in the extracted dataset and were not consistently available. Therefore, the proportion of patients with short stature, stunting, or loss of adult height potential could not be reliably determined.

Statistical analysis

Data were entered into Microsoft Excel (Microsoft Corporation, Redmond, Washington) and analyzed using IBM SPSS Statistics for Windows, Version 29 (Released 2023; IBM Corp., Armonk, New York). Descriptive statistics were used to summarize the study variables. Categorical variables were reported as frequencies and percentages. Continuous variables, including age at onset, age at diagnosis, and BMI, were summarized using mean, standard deviation, and range. Missing values were not imputed. No inferential statistical testing was performed because the primary purpose of the study was descriptive.

Ethical considerations

The study involved a retrospective review of the medical records of pediatric patients and was approved by the Institutional Review Board/Research Ethics Committee of King Fahd Hospital, Al-Hofuf Al Ahsa, under approval number 43-EP-2026. The requirement for individual informed consent was waived by the ethics committee because of the retrospective chart-review design and use of previously collected clinical information. Data were de-identified before analysis, and no directly identifiable patient information was reported.

## Results

Table 1 presents the baseline demographic, auxological, clinical, diagnostic, and management characteristics of the 87 included patients. The mean age at diagnosis was 7.4 ± 2.1 years, with a range of 4-16 years, while the mean age at pubertal onset was 6.2 ± 1.9 years, with a range of 2-8 years. Eligibility was based on the age at onset of pubertal development rather than the age at formal diagnosis; therefore, the upper diagnostic age reflects delayed diagnosis or referral among patients whose documented pubertal onset occurred within the predefined precocious-puberty age threshold.

**Table 1:** Baseline Demographic, Auxological, Clinical, Diagnostic, and Management Characteristics of Children With Precocious Puberty (N = 87)

Variable	Category	n (%) / Mean ± SD / Range
Age at diagnosis, years	Mean ± SD	7.4 ± 2.1
	Range	4–16
Age at onset, years	Mean ± SD	6.2 ± 1.9
	Range	2–8
Age group at diagnosis	<6 years	24 (27.6)
	6–7.9 years	29 (33.3)
	≥ 8 years	34 (39.1)
Sex	Female	74 (85.1)
	Male	13 (14.9)
BMI, kg/m²	Mean ± SD	21.6 ± 5.8
	Range	11.5–42.3
BMI category	Underweight, <5th percentile	5 (5.7)
	Normal, 5th–84th percentile	33 (37.9)
	Overweight, 85th–94th percentile	24 (27.6)
	Obese, ≥95th percentile	25 (28.7)
Family history of precocious puberty	Yes	6 (6.9)
	No	81 (93.1)
Parental consanguinity	Yes	19 (21.8)
	No	68 (78.2)
Type of precocious puberty	Central precocious puberty	82 (94.3)
	Peripheral precocious puberty	5 (5.7)
Clinical pubertal features at presentation	Breast development	74 (85.1)
	Pubic hair development	61 (70.1)
	Testicular enlargement	13 (14.9)
	Accelerated growth velocity	56 (64.4)
Bone age status	Advanced	36 (41.4)
	Normal	18 (20.7)
	Not documented	33 (37.9)
Brain MRI status	Normal	59 (67.8)
	Abnormal findings	7 (8.0)
	Not performed / not documented	21 (24.1)
Management approach	GnRH analogue therapy	83 (95.4)
	Observation / no intervention	4 (4.6)

The mean BMI was 21.6 ± 5.8 kg/m². Based on BMI-for-age percentiles, five patients (5.7%) were underweight, 33 (37.9%) had a healthy-weight BMI, 24 (27.6%) were overweight, and 25 (28.7%) had obesity. Overall, 49 patients (56.3%) were overweight or obese. Height-for-age z-scores and final-height outcomes were not available as predefined study variables; therefore, the prevalence of short stature or linear-growth impairment could not be determined from the available dataset.

Based on the final diagnoses documented in the pediatric endocrinology records, CPP was recorded in 82 patients (94.3%) and peripheral precocious puberty in five patients (5.7%). Individual hormonal assay values were not consistently available across the retrospective dataset and consequently are not presented as summary laboratory measures. This diagnostic classification should therefore be interpreted as the documented clinical diagnosis rather than an independent biochemical reclassification performed for the present study.

Table 2 summarizes the brain MRI findings among the study cohort and among patients who underwent imaging. Brain MRI was performed or documented in 66 patients. Among the total cohort, MRI was normal in 59 patients (67.8%) and abnormal in seven patients (8.0%), while MRI was not performed or documented in 21 patients (24.1%). Among patients who underwent MRI, abnormal findings were identified in seven of the 66 patients (10.6%). The abnormal MRI findings were heterogeneous. Congenital or developmental CNS abnormalities were documented in two patients, acquired ischemic, hemorrhagic, or traumatic encephalomalacic changes were documented in two patients, hydrocephalus or shunt-related abnormality was documented in one patient, post-surgical arachnoid cyst-related changes were documented in one patient, and borderline pituitary size without a discrete lesion was documented in one patient. Importantly, no hypothalamic hamartoma, pituitary adenoma, suprasellar mass, or other intracranial mass lesion was identified.

**Table 2:** Brain MRI Findings Among Children With Precocious Puberty

Brain MRI status/category	n (%) of total cohort, N = 87
MRI status	
Normal MRI	59 (67.8)
Abnormal MRI	7 (8.0)
MRI not performed / not documented	21 (24.1)
Type of abnormal MRI finding, n = 7	
Congenital/developmental CNS abnormality	2 (2.3)
Hydrocephalus or shunt-related abnormality	1 (1.1)
Acquired ischemic/hemorrhagic/traumatic encephalomalacic changes	2 (2.3)
Post-surgical arachnoid cyst-related change	1 (1.1)
Borderline pituitary size without discrete lesion	1 (1.1)

Figure 1 demonstrates the distribution of brain MRI findings in the study cohort. Brain MRI was normal in 59 patients (67.8%), abnormal in seven patients (8.0%), and not performed or not documented in 21 patients (24.1%). This indicates that most imaged patients had no abnormal MRI findings.

**Figure 1:**
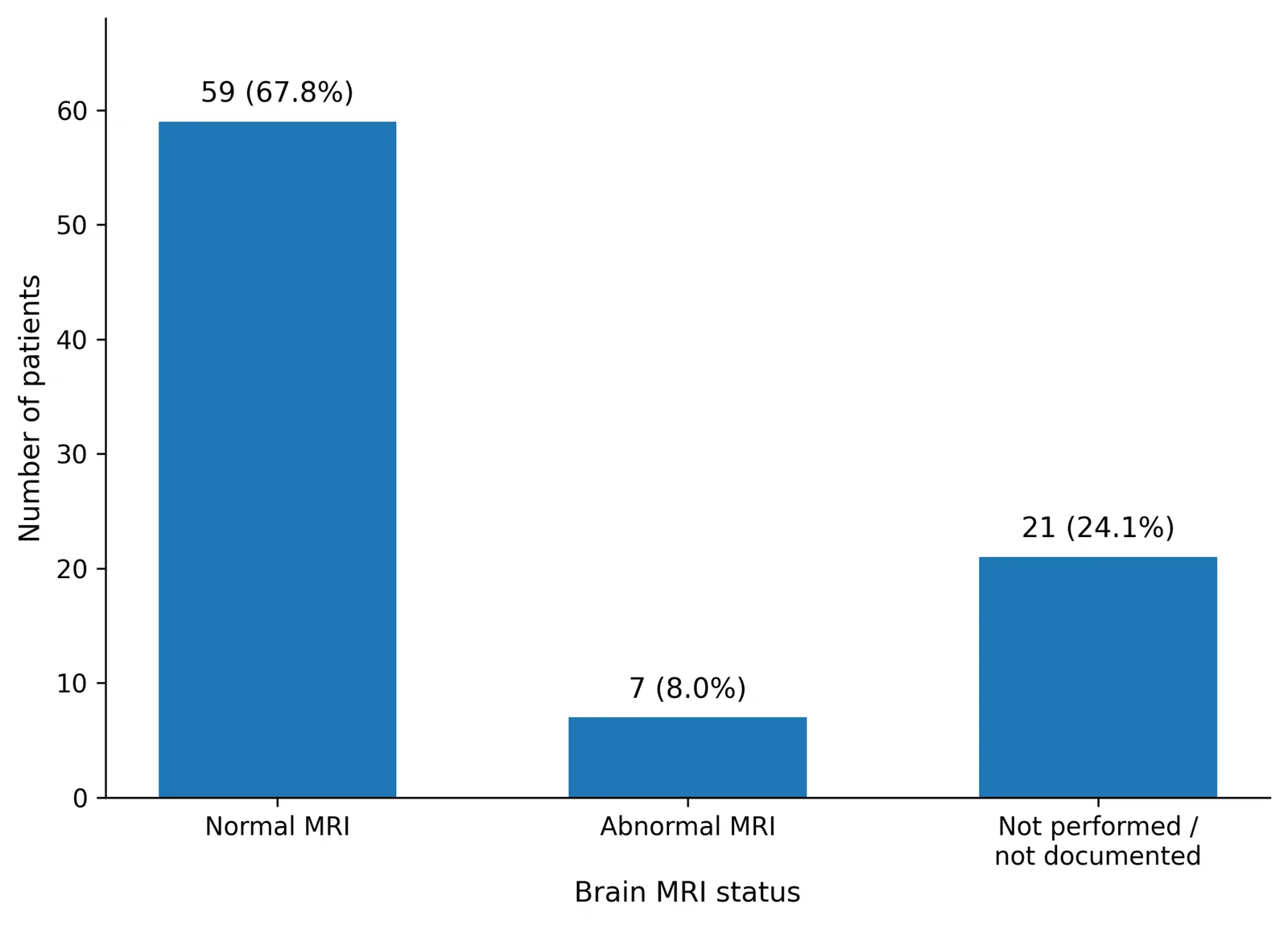
Distribution of Brain MRI Findings Among Children With Precocious Puberty

## Discussion

This retrospective study provides a descriptive profile of children with precocious puberty evaluated at a pediatric endocrinology clinic in Al Ahsa, Saudi Arabia. The principal findings were a marked female predominance and a predominance of documented CPP, which are broadly consistent with previous Saudi and international reports [1,3,11]. Overweight and obesity were also common, while most patients who underwent brain MRI had normal findings. Because the study was based on retrospective clinical records, the reported diagnostic classifications and management patterns reflect documented routine clinical practice rather than independent prospective validation of individual diagnostic or treatment decisions.

The predominance of CPP in the present cohort is consistent with the established epidemiology of true precocious puberty, particularly among girls [1,3]. Previous Saudi data from a tertiary center similarly demonstrated a predominance of girls and CPP among children presenting with precocious puberty [11]. Peripheral precocious puberty was uncommon in our cohort but remains clinically important because it may result from adrenal, gonadal, thyroid, genetic, neoplastic, or exogenous causes [1,3]. Biochemical assessment, including measurement of gonadotropins and sex steroids and the use of stimulation testing when clinically indicated, remains an important component of confirming hypothalamic-pituitary-gonadal axis activation and distinguishing CPP from other causes of early pubertal development [16,17]. Importantly, individual basal and stimulated gonadotropin and sex-steroid concentrations were not consistently available for retrospective extraction in the present study. Therefore, the study could not independently reapply uniform biochemical criteria to every patient, and the reported CPP and peripheral precocious puberty classifications represent the final diagnoses documented by the treating pediatric endocrinologists after integration of the available clinical, biochemical, radiological, and bone age assessments. This should be considered when interpreting the high proportion of patients classified as CPP.

The mean age at pubertal onset was lower than the mean age at formal diagnosis, suggesting that delayed specialist evaluation or diagnosis occurred in at least some patients. Eligibility for the present study was based on the documented age at pubertal onset rather than the age at eventual diagnosis, which also explains why some patients were diagnosed considerably later despite meeting the age criteria for precocious puberty at onset. Delayed evaluation of progressive precocious puberty may allow continued skeletal maturation and potentially compromise adult height potential [1,5,6]. However, height-for-age z-scores, predicted adult height, and final adult height were not systematically collected in the present study. Consequently, the proportion of patients with short stature, stunting, or compromised final height could not be determined, and no conclusions regarding the magnitude of linear-growth impairment can be drawn from this cohort.

Excess body weight was common, with more than half of the cohort classified as overweight or obese. Obesity has been associated with earlier pubertal timing, particularly among girls [8,9]. Several mechanisms have been proposed, including alterations in leptin signaling, insulin resistance, adrenal androgen production, and peripheral aromatization of androgens to estrogens [8,9]. Nevertheless, the retrospective descriptive design of the present study does not permit causal inference. The high frequency of overweight and obesity should therefore be interpreted as a coexisting clinical characteristic rather than evidence that excess adiposity caused precocious puberty in these patients.

Advanced bone age was documented in 41.4% of the total cohort and in 66.7% of patients with an available bone age assessment. Bone age evaluation is clinically useful in children with suspected precocious puberty because sex-steroid exposure may accelerate skeletal maturation and because the degree of advancement can contribute to assessment of disease progression and remaining growth potential [1,3-5,16]. Interpretation of our findings is limited, however, by the absence of documented bone age assessment in 37.9% of the cohort. Furthermore, the specific bone age assessment method and numerical threshold used to define advancement were not consistently recorded in the retrospective medical records. The observed proportion of advanced bone age should therefore be interpreted in the context of these documentation limitations rather than as a standardized estimate across the entire cohort.

Brain MRI was abnormal in 10.6% of patients who underwent imaging. The documented abnormalities were heterogeneous and included congenital or developmental CNS abnormalities, hydrocephalus or shunt-related changes, acquired encephalomalacic changes, post-surgical arachnoid cyst-related changes, and borderline pituitary size without a discrete lesion. No hypothalamic hamartoma, pituitary adenoma, suprasellar mass, or other intracranial mass lesion was identified. Neuroimaging remains an important component of the evaluation of selected patients with CPP, particularly when the age at onset, sex, neurological findings, or other clinical features increase concern for an underlying CNS lesion [1,3,16,17]. In this retrospective cohort, MRI was performed according to routine clinical judgment rather than a study-specific imaging protocol, and MRI was not performed or documented in 24.1% of patients. Therefore, the MRI findings should not be interpreted as an estimate of the prevalence of CNS abnormalities among all children with precocious puberty.

Most patients in the study received GnRH analogue therapy. GnRH analogues are the established treatment for appropriately selected children with progressive CPP, with the principal goals of suppressing premature hypothalamic-pituitary-gonadal axis activation, slowing pubertal progression, and preserving height potential [4-6,16]. However, the high treatment proportion in this cohort should not be interpreted as an independent assessment of treatment appropriateness. Treatment decisions were made by the treating pediatric endocrinologists during routine clinical care, and complete baseline hormonal assay results were not consistently available for retrospective analysis. The high treatment rate may also reflect referral bias, as patients evaluated in a specialized pediatric endocrinology clinic are more likely to have progressive or clinically significant disease than children with benign or nonprogressive early pubertal variants.

The present study should not be interpreted as a population-based prevalence study. Reported prevalence and incidence estimates of precocious puberty vary substantially across populations because of differences in diagnostic definitions, ascertainment methods, ethnicity, environmental exposures, obesity patterns, and healthcare access [8,9]. The strength of the present study lies instead in providing descriptive clinical data from Al Ahsa, where published information on precocious puberty remains limited. Previous Saudi literature has largely focused on pubertal timing, community awareness, or relatively small tertiary-center cohorts [11-14]. Our findings therefore add contemporary local information regarding the recorded clinical presentation, diagnostic evaluation, imaging findings, and management patterns of affected children.

Several limitations should be considered. First, the retrospective design made the study dependent on the completeness and accuracy of existing medical records and introduced the possibility of selection bias, information bias, and unmeasured confounding. Second, the study was conducted at a single specialized center and included a relatively small cohort, particularly only 13 male patients and five patients with peripheral precocious puberty, limiting evaluation of sex-specific differences and uncommon clinical manifestations and reducing generalizability. Third, individual basal and stimulated hormonal assay results were not consistently available in an extractable format, preventing independent retrospective biochemical confirmation of the documented CPP and peripheral precocious puberty classifications. Fourth, diagnostic information was incomplete for a substantial proportion of patients; bone age was not documented in 37.9% of cases, and brain MRI was not performed or not documented in 24.1%. In addition, the bone age assessment method, numerical threshold for defining advancement, and criteria for obtaining brain MRI were not standardized for research purposes because these investigations had been performed as part of routine clinical care. Finally, height-for-age z-scores, predicted and final adult height, treatment duration, biochemical response to GnRH analogue therapy, menarche timing, and psychosocial outcomes were not systematically available. These limitations restrict assessment of long-term growth and treatment outcomes and should be considered when interpreting the study findings.

Despite these limitations, the study contributes local clinical data on a relatively uncommon pediatric endocrine condition in Saudi Arabia. The findings support the need for more standardized documentation of diagnostic biochemical parameters, bone age assessment, imaging indications, and longitudinal growth outcomes. Larger prospective multicenter studies incorporating standardized hormonal testing and long-term height and treatment outcomes would provide a more comprehensive understanding of precocious puberty among children in Saudi Arabia.

## Conclusions

In this single-center retrospective cohort, the diagnoses recorded in the medical records were predominantly CPP and occurred mainly among girls. Overweight and obesity were common, while most patients who underwent brain MRI had no abnormal imaging findings. Advanced bone age was frequently documented among patients with available bone age assessment. Because individual diagnostic hormonal assay values and longitudinal height outcomes were not consistently available, the findings should be interpreted as a descriptive profile of documented clinical diagnoses and management rather than as an independent biochemical validation of case classification or an assessment of final growth outcomes. The study provides additional clinical data from Al Ahsa and supports the need for standardized diagnostic documentation and larger multicenter studies with complete hormonal and longitudinal growth data.
